# Identification of Nutritional Factors to Evaluate Periodontal Clinical Parameters in Patients with Systemic Diseases

**DOI:** 10.3390/nu15020365

**Published:** 2023-01-11

**Authors:** Yohei Nakayama, Shinichi Tabe, Arisa Yamaguchi, Yuto Tsuruya, Ryoki Kobayashi, Katsunori Oyama, Daisuke Kitano, Keisuke Kojima, Rikitake Kogawa, Yasuo Okumura, Jun Ogihara, Hidenobu Senpuku, Yorimasa Ogata

**Affiliations:** 1 Department of Periodontology, Nihon University School of Dentistry at Matsudo, Chiba 271-8587, Japan; 2 Research Institute of Oral Science, Nihon University School of Dentistry at Matsudo, Chiba 271-8587, Japan; 3 Department of Microbiology and Immunology, Nihon University School of Dentistry at Matsudo, Chiba 271-8587, Japan; 4 Department of Computer Science, College of Engineering, Nihon University, Fukushima 963-8642, Japan; 5 Division of Cardiology, Department of Medicine, Nihon University School of Medicine, Tokyo 173-8610, Japan; 6 Laboratory of Applied Microbiology and Biotechnology, College of Bioresource Sciences, Nihon University, Fujisawa-shi 252-0880, Japan

**Keywords:** chronic periodontitis, nutritional factors, systemic disease, correlation coefficients, multiple linear regression

## Abstract

Nutritional factors reflect the periodontal parameters accompanying periodontal status. In this study, the associations between nutritional factors, blood biochemical items, and clinical parameters were examined in patients with systemic diseases. The study participants were 94 patients with heart disease, dyslipidemia, kidney disease, or diabetes mellitus. Weak negative correlation coefficients were found between nine clinical parameters and ten nutritional factors. Stage, grade, mean probing depth (PD), rate of PD 4–5 mm, rate of PD ≥ 6 mm, mean clinical attachment level (CAL), and the bleeding on probing (BOP) rate were weakly correlated with various nutritional factors. The clinical parameters with coefficients of determinations (R^2^) > 0.1 were grade, number of teeth, PD, rate of PD 4–5 mm, CAL, and BOP rate. PD was explained by yogurt and cabbage with statistically significant standardized partial regression coefficients (yogurt: −0.2143; cabbage and napa cabbage: −0.2724). The mean CAL was explained by pork, beef, mutton, and dark green vegetables with statistically significant standardized partial regression coefficients (−0.2237 for pork, beef, and mutton; −0.2667 for dark green vegetables). These results raise the possibility that the frequency of intake of various vegetables can be used to evaluate periodontal stabilization in patients with systemic diseases.

## 1. Introduction

Chronic periodontitis is one of the most common causes of tooth loss, the progression of which depends on a balance between periodontopathic bacteria and the host immune response [1,2]. Tooth loss is directly linked to mastication ability and an increasingly evident increase in the patient’s systemic pathological state. Mastication ability partially influences unbalanced dietary habits [3,4]. Therefore, chronic periodontitis is increasingly recognized as a major public health problem, and chronic inflammation and lifestyle diseases continue to affect populations around the world.

Evaluations of periodontal status are mainly classified according to three factors, namely, the destruction of periodontal tissue, inflammation, and risk factors; therefore chronic periodontitis is recognized as a multifactorial lifestyle-related disease [5]. Deteriorated periodontal tissue leads to intermittent or continuous immune responses over time, such as changes in the clinical attachment level (CAL), probing depth (PD), and periodontal epithelial surface area (PESA) [6]. Inflammation is evaluated by bleeding on probing (BOP), which indicates inflammatory activity [7]. The risk factors for periodontitis mainly include smoking, the Brinkman (Br) Index [8], stress, and nutritional factors. However, standard nutritional factors have not been definitively unestablished for periodontal risk evaluations.

In the presymptomatic state, which is said to be the interlevel between a healthy and a disease condition, the functionality of food is considered to be more useful than medication in terms of treatment. A similar gap may exist between the progression of chronic periodontitis and a stable condition. Nuclear receptors have been reported to be the mechanism underlying the functionality of food, such as vitamin A [9]. Various metabolites derived from nutrients have been regarded as mere intermediates; however, they may perform various functions such as signal repair and epigenetic regulation via metabolites [10]. In addition, it has been noted that the intestinal microbiome plays an important role in metabolic changes and the functionality of food [11]. Moreover, it has been reported that the oral administration of *Lactobacillus gasseri* SBT2055 (LG2055)-rich yogurt inhibits the destruction of periodontal tissue via a reduction of inflammatory cytokines in *Porphyromonas gingivalis*-infected mice [12]. *Lactobacillus helveticus* SBT2171 (LH2171) is known to upregulate β-defensin production and reduce *P. gingivalis*-induced proinflammatory cytokine expression in gingival epithelial cells [13]. These previous studies support the hypothesis that nutritional factors affect periodontal stabilization either directly in the oral region or through interactions with the gut microbiome [14].

Close links have been reported between periodontal disease and nutritional status, including vitamin A [15], vitamins C and E, calcium [16], carotenoids, which have antioxidant effects, and calcium and magnesium, which are associated with bone metabolism [17]. Our previous study showed that the intake frequencies of dark green vegetables and yogurt influenced periodontal status, as confirmed by statistical analysis between questionnaire scores for nutritional factors and clinical parameters in patients with periodontitis undergoing supportive periodontal therapy (SPT) [18]. The results suggested that β-carotene antioxidants and changes in the gut microbiome after yogurt consumption affect periodontal status. However, the participants with systemic diseases were not objected in the study. Therefore, the relationships were unclear between periodontal clinical parameters and nutritional factors, and systemic disease.

Associations have been reported between periodontitis and systemic diseases, such as ischemic cardiac disease, dyslipidemia, chronic kidney disease (CKD), and diabetes mellitus (DM). Multivariate logistic regression models have revealed statistically significant differences between periodontitis and ischemic cardiac disease, including arteriosclerosis, angina pectoris, and myocardial infarction (males: odds ratio [OR] = 1.51, 95% confidence interval [CI]: 0.90–2.52; females: OR = 1.48, 95% CI: 0.95–2.32) [19]. On the contrary, a systematic review found no associations between morbidity associated with periodontitis and the onset of ischemic cardiac disease (hazard ratio [HR]: 1.14, 95% CI: 0.96–1.36) [20]. In a meta-analysis, a random effects model showed that PD increased the odds of dyslipidemia by 15% (OR = 1.15, 95% CI: 1.04–1.26), suggesting that periodontitis is associated with an increased risk of dyslipidemia [21]. These findings were supported by an investigation of the association between the severity of periodontitis and dyslipidemia, which reported a higher prevalence of dyslipidemia in moderate and severe periodontitis compared with non-periodontitis [22]. A few studies have examined the association between periodontitis and CKD, and periodontitis and kidney diseases are considered to worsen together. A 4-year cohort study reported that CKD was associated with significantly higher odds of CAL progression (adjusted OR = 1.73; 95% CI: 1.15–2.60) [23]. The OR of CKD for participants in the highest quartile of serum antibody to *P. gingivalis* was 2.59 (95% CI: 1.05–6.34) compared with others in lower quartiles [24]. Moreover, the highest periodontal inflamed surface area (PISA) quartile was significantly associated with a greater cumulative incidence of decreased kidney function (OR = 2.24, 95% CI: 1.05–4.79) compared with the referent group (the other three quartiles) during a 2-year follow-up [25]. Regarding the mutual association between periodontitis and DM, numerous studies have been conducted [26,27,28]. In a recent study, a multivariable-adjusted model demonstrated that the presence (vs. absence) of periodontitis was associated with a 66% increased risk of diabetes (OR = 1.66, 95% CI: 1.43–1.94); in addition, the risk of diabetes was much higher among those with severe (OR = 2.31, 95% CI: 1.72–3.11) compared with moderate periodontitis (OR =1.54, 95% CI: 1.30–1.82) [21]. A meta-analysis demonstrated that the risk of DM showed a gradient increase by severity of periodontitis (moderate relative risk [RR] = 1.20, 95% CI: 1.11–1.31; severe RR = 1.34, 95% CI: 1.10–1.63) [29]. Such statistical studies provide further evidence for the close relationship between these systemic diseases and periodontitis. Patients with these systemic diseases receive instruction in regard to their dietary habits [30,31,32,33]; therefore, these instructions may influence the association between periodontal parameters and nutritional factors. However, our previous study demonstrated that the frequency of yogurt and dark green vegetable consumption had positive effects on periodontal stabilization [18].

Given this background, the present study aimed to clarify the association between periodontal parameters and nutrient intake in patients with systemic diseases by conducting a questionnaire survey on lifestyle-related factors, including nutrient intake, and carrying out periodontal examinations.

## 2. Materials and Methods

### 2.1. Subjects

Patients who had undergone medical therapy for ischemic heart disease (e.g., angina, myocardial infarction), dyslipidemia (e.g., hypercholesterolemia), kidney disease (e.g., chronic nephritis), or DM were recruited from the department of cardiovascular medicine at the Nihon University School of Medicine Hospital. The research contents were explained to patients, and all patients who provided consent participated in this study. The exclusion criteria for analysis were as follows: (1) an edentulous jaw, (2) an insufficient amount of saliva for enzyme-linked immunosorbent assay (ELISA). Profiling of periodontal status and diseases was shown (Table 1).

### 2.2. Periodontal Examinations

In the periodontal examinations, the number of remaining teeth, teeth mobility, PD, BOP [7], plaque control records [34], and gingival recession were examined. All measurements were performed by one tester (Y.N.) who is a board-certified doctor (BD) from the Japanese Society of Periodontology. Therefore, calibration was not carried out same as in a previous study [35]. CAL was calculated based on PD, gingival recession (GE), PESA [6], and PISA, PISA/PESA was also calculated from the recorded data. Eichner’s classification was converted into numerals from the records, the same as in a previous study [36].

### 2.3. Questionnaire on Lifestyle Habits and Collection of Patient Information

A questionnaire survey was also conducted using a semi-quantitative food frequency questionnaire. The dietary assessments consisted of environmental factors associated with periodontitis, including smoking status (current smoker, former smoker, nonsmoker) and their durations, and the basic items included age, sex, height, and weight. The Br index and body mass index (BMI) [37] were calculated from the data. The items in the nutritional questionnaire, including dietary habits, were selected and modified based on the 2017 National Health and Nutrition Survey in Japan (Table S1) [18]. The questionnaires were self-reported by the participants. Information on prescription medicines was recorded from electronic clinical records, and dental histories were obtained from self-reports.

### 2.4. Analysis of the Association between Periodontal Clinical Parameters and Environmental and Nutritional Factors

In addition to four environmental factors (age, smoking, Br index, and BMI), nutritional factors (23 items) (Tables S1–S3) and clinical parameters (11 items) (Table 2) were used for the statistical analysis. Spearman’s rank correlation coefficients between clinical parameters and environmental and nutritional factors were calculated. Four environmental and 10 nutritional factors that had correlation coefficients |***r_s_***| > 0.15 with clinical parameters were used as explanatory variables in multiple regression analysis.

### 2.5. Enzyme-Linked Immunosorbent Assay (ELISA)

To investigate inflammatory status in saliva, protein levels were analyzed by ELISA. Approximately 3.0–4.0 mL of non-stimulated saliva was collected from the patients into specialized containers (Saliva Collection Aid Cryogenic Vials; 5016.02, Funakoshi Co., Ltd., Tokyo, Japan) during the periodontal examinations and then immediately frozen. Interleukin-1β (IL1β) protein levels were measured by an ELISA kit (human IL1β/IL1F2, DLB50; R&D Systems, Minneapolis, MN, USA). The procedures were carried out according to the manufacturer’s instructions. The data were used to examine the correlation with periodontal clinical parameters and environmental and nutritional factors to support the evaluation of oral inflammation.

### 2.6. Statistical Analysis

Spearman’s rank correlation coefficient (***r_s_***) was used to analyze the correlations between clinical parameters and environmental and nutritional factors, as well as between IL1β levels and environmental, nutritional, and clinical parameters.

Multiple regression analysis was conducted to clarify the causal dependencies of the correlation coefficients (|***r_s_***|> 0.15) between clinical parameters and environmental and nutritional factors, and to derive standardized partial regression coefficients, adjusted multiple correlation coefficients (R), adjusted coefficients of determination (R^2^), and regression variation. A variance inflation factor (VIF) < 10 and tolerance > 0.1 were used to confirm the absence of linear combinations between the explanatory variables. The stepwise method was used for variable selection in the multiple regression analysis. To determine whether autocorrelations existed between the error terms (the differences between the measured values and theoretical values), Durbin–Watson ratios |***r***|< 2.0 were confirmed. To evaluate the accuracy of the multiple linear regression analysis, mean absolute errors (MAEs) were calculated. Residual plots were generated to evaluate regularity and distribution visually, and the Breusch–Pagan and White tests were used to evaluate heteroskedasticities.

According to the results of the multiple regression analysis, six clinical parameters (number of teeth, PD, CAL, BOP rate, PISA, PESA, and PISA/PESA) were selected and stratified by the clinical standard [6,38], and then stratified descriptive statistics were performed between the clinical parameters and environmental and nutritional factors. The sample sizes were confirmed using the effect size (dose: 0.873) that calculated Yogurt score in evaluating the frequency by same guide (Table S1), an alpha risk of 0.05 in a two-sided test by software (G*Power 3.1.9.7) [18]. The calculated sample size was over 29 subjects in each group. For the analysis of environmental factors, number of teeth, BOP rate, PISA, PESA, and PISA/PESA were selected, whereas for the analysis of nutritional factors, number of teeth, PD, and CAL were selected. The correlation ratio (η^2^) and testing of differences of the population mean were then calculated. Next, *t*-tests or Welch’s *t*-tests were selected according to the statistical normality and equal variance of the variables, and used to compare differences of the population mean. A *p* value < 0.05 was considered to indicate statistical significance. 

## 3. Results

### 3.1. Profiling of Periodontal Examinations and Environmental and Nutritional Factors

A total of 101 patients took part in the collection of saliva, lifestyle questionnaire, and periodontal examinations. All blood biochemical tests were conducted on the same day before the periodontal examinations. In total, seven patients were excluded from the analysis because the amount of saliva collected was insufficient to analyze oral inflammation by ELISA. Finally, 94 patients were included in the study and diagnosed based on blood biochemical test standards. Among these 94 patients, 49 were diagnosed with heart disease, 20 with dyslipidemia, 35 with kidney disease, and 28 patients with DM. A total of 49 patients had visited a dentist for treatment or SPT within the preceding 6 months. The rates of dental visits and periodontal statuses (extent, stage, and grade) were similar between the systemic disease subgroups (Table 1), which indicated that neither dental history, interventions, nor periodontal status affected the results of the statistical analyses of differences between nutritional factors.

The results of the questionnaires on lifestyle habits regarding nutritional and environmental factors are shown in Table S2, and a frequency table is shown in Table S3 [18]. Histograms close to the normal distribution were seen for most of the nutritional factors; however, rice was concentrated around scores 6 and 7. Meanwhile, liver and squid, octopus, shrimp, and shellfish were concentrated around scores 1 and 2, and egg, milk, yogurt, and dark green vegetables demonstrated bimodality (Figure S1). Spearman’s rank correlation coefficients between items regarding the blood biochemical examinations and nutritional factors showed some consistencies, such as weak negative correlations between bread and aspartate aminotransferase (AST) (***r_s_*** = −0.269), milk and ALT (***r_s_*** = −0.216), pork, beef, and mutton (***r_s_*** = −0.257), other vegetables (***r_s_*** = −0.252), and creatine kinase (CK) in patients with heart disease, and weak positive correlations between pork, beef, and mutton (***r_s_*** = 0.218), cabbage and napa cabbage (***r_s_*** = 0.229), radishes and turnips (***r_s_*** = 0.225), and total cholesterol (T-cho) in patients with dyslipidemia, and processed meat and hemoglobin A1c (HbA1c) (***r_s_*** = 0.225) in patients with DM (Table S4). Despite the fact that the patients in this study received instructions regarding dietary habits from specialists in cardiovascular medicine, some poor dietary habits remained.

### 3.2. Correlation Coefficient Matrix between Clinical Parameters and Environmental and Nutritional Factors

The correlation coefficients (at least two items of 0.15 < |***r_s_***|) were demonstrated between 11 clinical parameters, four environmental factors, and 10 nutritional factors (Table 2). Among the environmental factors, weak negative correlations were found between age and number of teeth (***r_s_*** = −0.279), PISA (***r_s_*** = −0.291), and PESA (***r_s_*** = −0.376). On the other hand, weak positive correlations were found between smoking and grade (***r_s_*** = 0.226), rate of PD 4−5 mm (***r_s_*** = 0.309), BOP rate (***r_s_*** = 0.277), PISA (***r_s_*** = 0.291), PESA (***r_s_*** = 0.217), and PISA/PESA (***r_s_*** = 0.264). Regarding nutritional factors, weak positive correlations were found between number of teeth and pork, beef, and mutton (***r_s_*** = 0.234), dark green vegetables (***r_s_*** = 0.232), and mushrooms (***r_s_*** = 0.335), and between PESA and mushrooms (***r_s_*** = 0.224), which may have been influenced by mastication ability. On the contrary, most of the nutritional factors showed negative weak correlations with stage, grade, PD, rate of PD 4−5 mm, CAL, BOP rate, PISA, and PISA/PESA. These results suggest that insufficient intake of vegetables and yogurt affects periodontal parameters.

### 3.3. Multiple Regression Analysis

Multiple regression analysis was performed using 11 clinical parameters as objective variables. Ten nutritional and three environmental factors were correlated with periodontal parameters, and these were utilized as explanatory variables (Table 3 and Table 4). Adjusted multiple correlation coefficients (R) and adjusted coefficients of determination (R^2^) were calculated to confirm the correlations between the clinical parameters and environmental and nutritional factors. The clinical parameters with R^2^ > 0.1 were PISA and PESA, which were explained by age and smoking. In explaining PISA, the standardized partial regression coefficient of smoking (0.2616) was statistically significant. However, the standardized partial regression coefficient of age had a statistically significant negative value (−0.3591) for explaining PESA. These results might be the result of tooth loss due to aging.

All R^2^ indicated statistically significant differences when explained by nutritional factors; however, the clinical parameters with R^2^ > 0.1 were grade, number of teeth, PD, rate of PD 4–5 mm, CAL, and BOP rate (Table 3). In explaining grade and BOP rate, no standardized partial regression coefficients showed statistically significant differences. The number of teeth was explained by mushrooms, with a positive standardized partial regression coefficient (0.3355); this might be attributed to mastication ability due to the remaining number of teeth. Noodles, tofu, yogurt, and cabbage and napa cabbage explained PD with statistically significant standardized partial regression coefficients (yogurt: −0.2143; cabbage and napa cabbage: −0.2724). The rate of PD 4–5 mm was explained by noodles, yogurt and cabbage and napa cabbage, with statistically significant standardized partial regression coefficients (cabbage and napa cabbage: −0.2549), and CAL was explained by pork, beef, and mutton and dark green vegetables, with statistically significant standardized partial regression coefficients (pork, beef, and mutton: −0.2237; dark green vegetables: −0.2667). These results suggest that the frequency of intake of these vegetables affects the stability of periodontal status.

Tolerance and VIF indicated no strong collinearities. The MAEs of the clinical parameters were found to be absolute values, as calculated by subtracting the predicted from the actual values, and similar values were found between environmental and nutritional factors. Doubtful plots were confirmed in the residual plots by Breusch–Pagan and White heteroskedasticity tests in the multiple regression analysis. The doubtful outliers were shown in the analysis of number of teeth when the explanatory variables were environmental factors, and in the analysis of number of teeth, PD, rate of PD 4–5 mm, rate of PD > 6 mm, and PESA when the explanatory valuables were nutritional factors. These values were not excluded from the analysis after confirming the adequacy of the clinical examinations (Figure S2 and Figure S3).

### 3.4. Stratified Descriptive Statistics between Clinical Parameters and Environmental and Nutritional Factors

**Figure 1:**
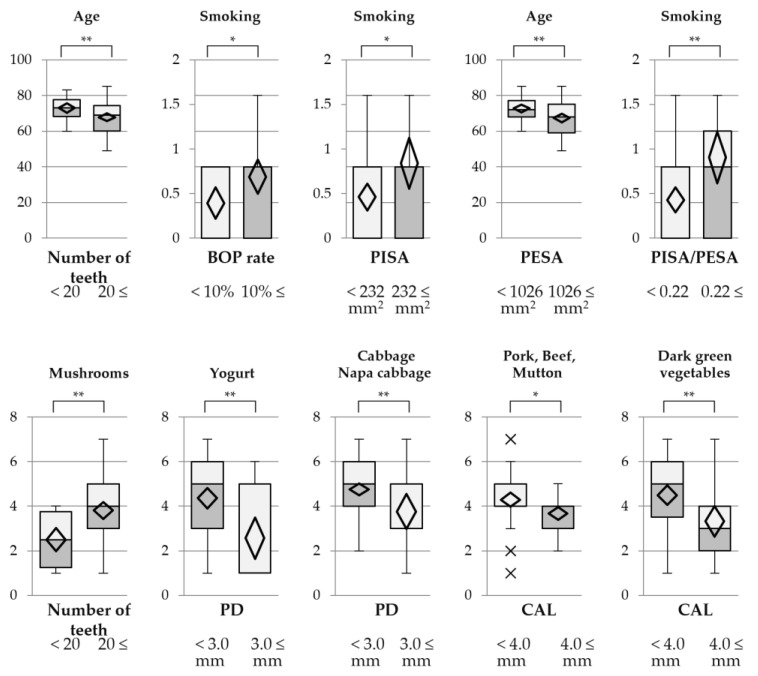
Box plot of environmental and nutritional factors between stratified groups in periodontal clinical parameters. Differences of population mean from clinical parameters were demonstrated by box plots in environmental and nutritional factors, respectively. The analysis that showed statistically significant differences in the results of stratified descriptive statistics were represent (Table 5 and Table 6). Abbreviations: PD, probing depth; CAL, clinical attachment level; BOP, bleeding on probing; PISA, periodontal inflamed surface area; PESA, periodontal epithelial surface area. The *t*-tests or Welch t-tests were used for comparisons of differences of population mean, respectively (* *p* < 0.05, ** *p* < 0.01).

Based on the results of the multiple regression analysis, a *t*-test or Welch’s *t*-test was conducted to examine the differences in environmental factors or frequency of nutrient intake between stratified groups of periodontal parameters (Table 5 and Table 6). Environmental and nutritional factors with statistically significant standardized partial regression coefficients were selected in this analysis. All objective periodontal clinical parameters were stratified into two groups based on clinical standards [6,38]. The patients with ≥ 20 teeth and PESA ≥ 1026 mm^2^ were significantly younger than those with <20 teeth and PESA < 1026 mm^2^. Current smokers tended to show inflammatory activity as reflected by the BOP rate, PISA, and PISA/PESA. The patients with PD < 3.0 mm showed significantly more frequent intakes of yogurt and cabbage and napa cabbage. The patients with CAL < 4.0 mm showed significantly more frequent intakes of pork, beef, and mutton and dark green vegetables (Figure 1). The correlations between these clinical periodontal parameters and factors were reevaluated by scatter diagrams, and tolerance distributions were confirmed by a few outliers (Figure S4). These results raised the possibility that frequent intakes of yogurt, cabbage and napa cabbage, pork beef, and mutton, and dark green vegetables are associated with the prevention of periodontitis.

### 3.5. Correlation Coefficients between Inflammatory Cytokines, Clinical Parameters, and Environmental and Nutritional Factors

Inflammatory status was evaluated based on IL1β protein levels in saliva. As a result, statistically significantly weak positive correlations were found between IL1β protein levels and PD (***r_s_*** = 0.381), rate of PD 4–5 mm (***r_s_*** = 0.271), rate of PD ≥ 6 mm (***r_s_*** = 0.317), CAL (***r_s_*** = 0.213), BOP rate (***r_s_*** = 0.353), PISA (***r_s_*** = 0.299), and PISA/PESA (***r_s_*** = 0.348). Statistically significantly weak positive correlations were also found between IL1β/bicinchoninic acid and number of teeth (***r_s_*** = 0.250), PD (***r_s_*** = 0.212), PISA (***r_s_*** = 0.258), PESA (***r_s_*** = 0.312), and PISA/PESA (***r_s_*** = 0.205). These results indicated that IL1β protein levels were reflected by clinical parameters (Table S6). However, IL1β protein levels were not correlated with blood biochemical items or environmental or nutritional factors (data not shown).

## 4. Discussion

There were several limitations in this study. First of all, there were many missing values in the blood examination data, since only clinically necessary blood items were examined for the patients who visited the cardiology department. They might cause missing values, which may have influenced variations in the calculation of correlation coefficients and comparisons of differences between groups. Secondary, some patients had multiple diseases, which may have affected nutritional factors and blood test data. Thirdly, since the responses to the nutritional factor questionnaire reflected the dietary statuses within one month, it was not clear whether they are appropriately reflected in the assessment of long-term nutritional and periodontal statuses.

A total of 94 patients who had been diagnosed with ischemic heart disease, dyslipidemia, CKD, or DM based on blood biochemical examinations participated in the present study. Numerous previous studies reported that these diseases were exacerbated by periodontitis; however, in the present study, no statistically significant differences were seen in the clinical parameters of periodontitis in the presence or absence of these diseases (Table 1). Approximately half of the patients had visited the dentist within the preceding 6 months, which may reflect the fact that they were receiving appropriate dental and periodontal treatments. In addition, no statistically significant differences were seen in any of the periodontal disease clinical parameters, blood test items, or nutritional factors between dental visits within/over 6 months (Table S5), confirming that the presence or absence of dental consultation was not a confounding factor in this study.

Next, we analyzed the correlation between blood test items and periodontal clinical parameters to confirm whether the frequency of nutrient intake showed different tendencies depending on the disease. Considering the negative relationships observed, it was suggested that higher CK levels were the result of insufficient intake of other vegetables, increased T-cho levels were due to the excessive intake of pork, beef, and mutton, and increased HbA1c levels were due to the excessive intake of processed meats (Table S4). These data reaffirmed the fact that these lifestyle-related diseases are closely involved with nutritional factors.

The correlation matrix between periodontal clinical parameters and nutritional factors showed many negative correlations (Table 2). In our previous study, we analyzed the correlations between eight periodontal clinical parameters and 10 nutritional factors, showing 18 negative correlation coefficients (***r_s_***≤ −0.200) in total [18]. In the analysis of the correlation coefficients between 11 periodontal disease clinical parameters and 10 nutritional factors in the present study, a total of 52 negative correlation coefficients (***r_s_*** ≤ −0.200) were found. These results suggested that nutritional factors were reflected in the clinical parameters of periodontal disease, even in patients with systemic diseases who received regular dietary guidance. In addition, positive correlations between smoking and periodontal disease clinical parameters were more pronounced in patients with than in those without systemic diseases [18]. Patients with systemic diseases or statuses tend to improve their lifestyle habits and refine their dietary intake and oral nutritional supplements [39]. Although beneficial, oral nutritional supplements (ONS) were concerned about their cariogenic potential [40]. It was unclear that ONS intake negatively and directly affects periodontal tissue condition. However, the ONS statuses should have been asked in this study.

Multiple regression analyses were performed using environmental and nutritional factors that were significantly correlated with periodontal clinical parameters (Table 3 and Table 4). Based on the results, stratified descriptive statistical analysis was performed (Table 5 and Table 6). The results indicated that smoking frequency and history differed depending on the BOP rate, PISA, and PISA/PESA.

The frequency of intake of yogurt and cabbage and napa cabbage stratified by PD were statistically significantly different (PD by nutritional factors: coefficient of determination R^2^ = 0.1256, standard partial regression coefficients with yogurt [−0.2143] and cabbage and napa cabbage [−0.2724]; correlation ratios with yogurt [η^2^ = 0.1226] and cabbage and napa cabbage [η^2^ = 0.0981]). Moreover, the frequency of intake of pork, beef, and mutton and dark green vegetables showed significant differences between the stratified CAL values (CAL by nutritional factors: R^2^ = 0.1334, standard partial regression coefficients with pork, beef, and mutton [−0.2237] and with dark green vegetables [−0.2667]; correlation ratios with pork, beef, and mutton [η^2^ = 0.0623] and dark green vegetables [η^2^ = 0.0897]) (Table 3 and Table 6, Figure 1).

Several studies have reported a positive interaction between intestinal bacterial flora and the oral environment [41,42], which emphasizes the medical importance of maintaining good health. It is also widely recognized that the intake of dairy products has positive effects, such as a reduced risk of DM, metabolic syndrome, and heart disease through improving the intestinal environment [43]. Individuals with a high intake of dairy products are less likely to have periodontitis [44,45], and the intake of yogurt was reported to help prevent tooth loss in a population-based prospective study [46]. Yogurt contains lactic acid bacteria, one of the main components of probiotics, which have been shown to be efficacious in periodontal therapy [47,48,49,50], although it should be noted that with respect to interpretation of these previous results, attention was needed in relation to the risk of bias in clinical trials [51]. Although these previous findings support the results of the present study, more evidence is needed to clarify the systemic and potential pathway from yogurt intake to PD.

Both cabbage and napa cabbage are rich in *S*-methylmethionine sulfonium (SMMS), a derivative of the amino acid methionine widely referred to as vitamin U because of its potent therapeutic effect on gastrointestinal ulceration [52]. The considerable activation of ERK1/2 by SMMS has been shown to promote the proliferation and migration of human dermal fibroblasts [53]. Although those previous studies demonstrated the direct and local effects of SMMS application, it was also reported that fresh cabbage juice accelerates rapid wound healing for peptic ulcers [54], raising the possibility that *S*-methylmethionine in cabbage has positive effects on periodontal stabilization. Meat is rich in vitamins B_6_ and B_12_, the adequate intake of which has been reported to improve collagen quality, which may be associated with the maintenance of gingival health. In addition, homocysteine, an essential amino acid and an intermediate product in methionine metabolism, has been shown to inhibit the formation of collagen structures, which are necessary for normal bone metabolism [55]. Therefore, adequate intake of vitamins B_6_ and B_12_, which reduce blood homocysteine levels, may have a positive effect on bone metabolism. It has been reported that carnosine is a functional component of meat, and has systemic effects such as the promotion of fat burning and anti-fatigue [56]. Studies involving periodontal tissue have reported that β-alanyl-l-histidine (carnosine) stimulates effusion at the initial stage of inflammation, thereby enhancing the wound healing process [57], as well as the expression of runt-related transcription factor-2/core binding factor alpha-1 (RUNX2/Cbfa1) and bone morphogenetic protein-2 (BMP-2) and BMP-7 in human periodontal ligament cells [58]. Carnosine concentrations in saliva were also reported to be significantly higher in patients with periodontitis than in healthy controls, suggesting the usefulness of carnosine as a periodontal biomarker [59].

It is well known that poor nutritional balance reduces the resistance of periodontal tissue to periodontal bacteria, and that deficiencies in carotenoids with antioxidant properties induce a weaker immune response [60]. In our previous study, the frequency of intake of dark green vegetables was negatively correlated with periodontal clinical parameters in non-prevalent patients [18]. The relevant periodontal clinical parameters indicated inflammatory activity, such as BOP and PISA, which was not consistent with the results of the present study. On the other hand, the association between yogurt and PD showed the same consistency regardless of the presence (***r_s_*** = −0.234, *p* < 0.05) or absence of systemic disease (***r_s_*** = −0.331, *p* < 0.01). These results suggest that the frequency of yogurt intake is the most useful of the nutritional factors for evaluating periodontitis. However further studies need to be performed by larger subjects, because some of their powers were slightly lower than 0.8 in comparison between Number of teeth < 20 and Number of teeth 20≤, PESA < 1062 and PESA 1062≤ (Figure 1).

We selected IL1β as an inflammation biomarker in this study, because IL1β mRNA levels demonstrated a stronger correlation with periodontal parameters and nutritional factors than TNFα in the previous study [18]. The positive correlation between IL1β protein concentrations in saliva and many periodontal disease parameters suggests its usefulness as a biomarker for understanding oral conditions (Table S6), as reported in the previous study [18]. However, in terms of the correlations between salivary IL1β protein concentrations and nutritional factors, only the frequency of carrot and squash intake showed a weak negative correlation (***r_s_*** = −0.154, data not shown). These results suggest that nutritional factors do not directly reflect salivary biomarker levels, although it is necessary to investigate the association between any other inflammatory biomarkers and nutrient habits.

## 5. Conclusions

The present results revealed that the frequency of intake of yogurt and cabbage and napa cabbage is useful in terms of nutritional factors for evaluating PD, and that the frequency of intake of pork, beef, and mutton and dark green vegetables is useful in the assessment of CAL, even in patients with systemic diseases. These results suggest that the frequent intake of yogurt products, cabbage and napa cabbage, pork, beef, and mutton, and dark green vegetables may help prevent periodontitis. Therefore, the evaluation of dietary habits should be added to the assessments of periodontal risk factors.

## Figures and Tables

**Table 1:** Profiling of periodontal status.

Objects		Total	Heart Disease		Dyslipidemia		Kidney Disease		Diabetes Mellitus	
			Heart Disease	Non	Dyslipidemia	Non	Kidney Disease	Non	Diabetes Mellitus	Non
	Total	94	49	45	20	74	35	59	28	66
Sex	Male	75	37	38	18	57	31	44	23	52
	Female	19	12	7	2	17	4	15	5	14
Age		70 (49−85)	69 (49−85)	71 (51−83)	72 (51−85)	70 (49−85)	73 (49−85)	69 (51−85)	71 (51−85)	69 (49−85)
Visit of dental clinic	Within 6 months	49	26	23	11	38	17	32	15	34
	Over 6 months	45	23	22	9	36	18	27	13	32
Periodontal status	Extent (localized/generalized)	84/10	44/5	40/5	18/2	66/8	28/7	56/3	25/3	59/7
	Stage (1/2/3/4)	10/24/37/23	5 /13/21/11	5/11/17/12	6/4/4/6	4/21/33/17	4/9/13/9	6/15/24/14	2/7/16/4	8/17/22/19
	Grade (A/B/C)	19/46/29	10 /21/ 18	9/25/11	5/9/6	14/37/23	6/16/13	13/30/16	5/13/10	14/33/19
Examination item	Number of teeth	25 (2−32)	25 (3−32)	25 (2−30)	24.5 (4−29)	25 (2−32)	25 (12−32)	24 (2−30)	26 (4−30)	24 (2−32)
	Median of PD (mm)	2.6 (2.1−5.2)	2.6 (2.1−5.0)	2.6 (2.1−5.2)	2.6 (2.1−3.7)	2.6 (2.1−5.2)	2.6 (2.1−5.2)	2.6 (2.1−5.0)	2.6 (2.1−3.7)	2.6 (2.1−5.2)
	Rate of PD 4–5 mm (%)	4.10 (0−50.0)	3.50 (0−50.0)	4.8 (0−39.5)	3.8 (0−30.1)	4.5 (0−50.0)	4.7 (0−50.0)	4.0 (0−25.0)	4.9 (0−38.7)	3.6 (0−50.0)
	Rate of PD ≥6 mm (%)	0 (0−32.5)	0 (0−31.7)	0.6 (0−32.5)	0.4 (0−16.7)	0 (0−32.5)	0.01 (0−32.5)	0 (0−31.7)	0.31 (0−16.7)	0 (0−32.5)
	Median of CAL (mm)	3.65 (2.3−8.3)	3.6 (2.3−6.6)	3.8 (2.4−8.3)	3.8 (2.3−6.6)	3.6 (2.4−8.3)	3.9 (2.4−8.2)	3.6 (2.3−8.3)	3.8 (2.6−5.7)	3.6 (2.3−8.3)
	BOP rate (%)	13.0 (1.3−63.0)	12.9 (1.3−63.0)	13.0 (1.8−42.3)	10.8 (1.3−42.3)	13.6 (1.7−63.0)	12.9 (1.3−63.0)	13.0 (1.3−61.7)	10.4 (1.3−41.7)	13.6 (1.3−63.0)
	PISA (mm^2^)	146.7 (7.1−1131.2)	154.4 (12.7−1131.2)	143.6 (7.1−915.4)	140.9 (12.7−915.4)	154.7 (7.1−1131.2)	154.4 (22.9−1131.2)	143.6 (7.1−660.0)	136.9 (12.7−680.7)	154.7 (7.1−1131.2)
	PESA (mm^2^)	1148.3 (132.7−2068.1)	1134.3 (132.7−2068.1)	1152.7 (137.3−2030.9)	1028.3 (246.3−1875.4)	1161.6 (132.7−2068.1)	1180.3 (637.8−2068.1)	1116.5 (132.7−1679.7)	1180.4 (246.3−2030.9)	1119.1 (132.7−2068.1)
	PISA/PESA	0.16 (0.012−0.710)	0.157 (0.012−0.710)	0.170 (0.015−0.488)	0.137 (0.012−0.488)	0.170 (0.015−0.71)	0.163 (0.024−0.609)	0.157 (0.012−0.71)	0.117 (0.012−0.484)	0.165 (0.015−0.71)
	PCR (%)	34.3 (3.6−98.1)	33.7 (3.6−98.1)	35.0 (6.0−88.0)	26.7 (3.6−73.1)	37.0 (6.0−98.1)	39.1 (7.7−97.2)	32.7 (3.6−98.1)	37.0 (7.7−77.6)	33.2 (3.6−98.1)
	Number of missing molars	4 (0−16)	4 (0−12)	4 (0−16)	4 (0−16)	4 (0−16)	4 (0−11)	4 (0−16)	3 (0−16)	4 (0−16)
	Classification of Eichner	4 (1−9)	4 (1−9)	4 (1−9)	4 (1−9)	4 (1−9)	4 (1−8)	4 (1−9)	4 (1−9)	4 (1−9)

The profiles of periodontal parameters recorded from the patients were demonstrated by four diseases (i.e., heart disease, dyslipidemia, kidney disease and diabetes mellitus). The numbers in each box indicated median (minimum—maximum). All periodontal statuses and examination items were not statistically significant differences between disease and non-diseases, respectively. Abbreviations: PD, probing depth; CAL, clinical attachment level; BOP, bleeding on probing; PISA, periodontal inflamed surface area; PESA, periodontal epithelial surface area; PCR, plaque control record.

**Table 2:** Correlation coefficient matrix between periodontal clinical parameters and environmental factors, nutritional factors.

	Environmental Factors				Nutritional Factors									
	Age	Smoking	Br Index	BMI	Noodle	Pork, Beef, Mutton	Processed Meat	Tofu	Yogurt	Dark Green Vegetables	Cabbage, Napa Cabbage	Carrot, Squash	Other Vegetables	Mushrooms
Stage	0.013	0.039	0.054	0.022	−0.114	−0.201 *	−0.038	−0.240 *	−0.155	−0.221 *	−0.214 *	−0.277 **	−0.200	−0.279 **
Grade	−0.095	0.226 *	0.227 *	0.034	−0.125	−0.208 *	−0.141	−0.213 *	−0.210 *	−0.312 **	−0.266 **	−0.332 **	−0.266 **	−0.259 *
Number of teeth (*n*)	−0.279 **	0.088	0.007	0.087	−0.051	0.234 *	0.005	0.160	0.116	0.232 **	0.146	0.101	0.056	0.335 **
PD (mm)	−0.102	0.101	−0.061	0.028	−0.165	−0.160	−0.229 *	−0.283 **	−0.331 **	−0.320 **	−0.408 **	−0.228 *	−0.225 *	−0.162
Rate of PD 4–5 mm (%)	−0.049	0.309 **	0.032	0.064	−0.203 *	−0.174	−0.156	−0.188	−0.268 **	−0.203	−0.333 **	−0.119	−0.039	−0.069
Rate of PD ≥6 mm (%)	−0.017	−0.124	−0.149	−0.102	−0.099	−0.064	−0.204 *	−0.230 *	−0.229 *	−0.257 *	−0.309 **	−0.211 *	−0.265 **	−0.165
CAL (mm)	0.191	0.040	0.042	−0.119	0.000	−0.351 **	−0.136	−0.204	−0.158	−0.325 **	−0.182	−0.145	−0.169	−0.294 **
BOP rate (%)	−0.188	0.277 **	0.077	0.091	−0.254 *	−0.127	−0.222 *	−0.240 *	−0.269 **	−0.223 *	−0.286 **	−0.177	−0.161	−0.079
PISA (mm^2^)	−0.291 **	0.291 **	0.082	0.129	−0.191	−0.070	−0.205 *	−0.191	−0.229 *	−0.130	−0.270 **	−0.153	−0.122	0.006
PESA (mm^2^)	−0.376 **	0.217 *	0.017	0.167	−0.118	0.099	−0.109	−0.028	−0.072	0.059	−0.073	−0.034	−0.049	0.224 *
PISA/PESA	−0.189	0.264 *	0.053	0.104	−0.256 *	−0.117	−0.211 *	−0.226 *	−0.257 *	−0.198	−0.265 **	−0.183	−0.166	−0.074
						*r_s_* > 0.20		*r_s_* < −0.25		*r_s_* < −0.20		*r_s_* < −0.15		

This panel showed Spearman’s rank-correlation coefficient (***r_s_***) between 10 periodontal clinical parameters and four environmental factors, 10 nutritional factors. Boxes in the matrix were painted by color in response to correlation coefficient as shown below. Student’s t distribution was used to compare differences among correlation coefficients (* *p* < 0.05, ** *p* < 0.01). Abbreviations: Br Index, Brinkman index; BMI, body mass index; PD, probing depth; CAL, clinical attachment level; BOP, bleeding on probing; PISA, periodontal inflamed surface area; PESA, periodontal epithelial surface area.

**Table 3:** Multiple regression analysis.

	Nutritional Factors							
Analysis Clinical Parameters	Correlation Coefficient |*r_s_*| > 0.15	Multiple Regression Analysis						
		*: *p* < 0.05 **: *p* < 0.01	Standardized Partial Regression Coefficient	Collinearity Statistics		R R^2^ Regression Variation	DW	MAE
				Tolerance	VIF			
Stage	•Processed meat •Yogurt •Dark green vegetable •Cabbage, Napa cabbage •Carrot, Squash •Other vegetables •Mushrooms	•Carrot, Squash •Mushrooms •Constant term **	−0.1712 −0.2239 -	0.6039 0.6039 -	1.6558 1.6558 -	0.2902 0.0842 *p* < 0.01	2.02	0.55
Grade	•Processed meat •Yogurt •Dark green vegeta-ble •Cabbage, Napa cab-bage •Carrot, Squash •Other vegetables •Mushrooms	•Dark green vegetables •Carrot, Squash •Constant term **	−0.1712 −0.2239 -	0.6039 0.6039 -	1.6558 1.6558 -	0.3294 0.1085 *p* < 0.01	1.81	0.55
Number of Teeth	•Pork, Beef, Mutton •Tofu •Dark green vegetables •Mushrooms	•Mushrooms ** •Constant term **	0.3355 -	1.0000 -	1.0000 -	0.3208 0.1029 *p* < 0.01	2.08	4.96
PD (mm)	All 10 nutritional factors	•Noodle •Tofu •Yogurt * •Cabbage, Napa cabbage * •Constant term **	−0.1356 −0.1344 −0.2143 −0.2724 -	0.9897 0.8510 0.8815 0.7777 -	1.0104 1.1750 1.1344 1.2858 -	0.4565 0.2084 *p* < 0.001	2.02	0.33
Rate of PD 4–5 mm (%)	•Noodle •Pork, Beef, Mutton •Processed meat •Tofu •Yogurt •Dark green vegetable •Cabbage, Napa cabbage	•Noodle •Yogurt •Cabbage, Napa cabbage * •Constant term **	−0.1796 −0.1827 −0.2549 -	0.9897 0.8866 0.8787 -	1.0104 1.1278 1.1380 -	0.3409 0.1162 *p* < 0.01	2.05	7.0
Rate of PD ≥6 mm (%)	•Processed meat •Tofu •Yogurt •Dark green vegetable •Cabbage, Napa cabbage •Carrot, Squash •Other vegetables •Mushrooms	•Cabbage, Napa cabbage * •Other vegetables •Constant term **	−0.2512 −0.1718 -	0.8311 0.8311 -	1.2033 1.2033 -	0.3151 0.0993 *p* < 0.01	2.07	2.96
CAL (mm)	•Pork, Beef, Mutton •Tofu •Yogurt •Dark green vegetable •Cabbage, Napa cabbage •Carrot, squash •Other vegetables	•Pork, Beef, Mutton * •Dark green vegetables ** •Constant term **	−0.2237 −0.2667 -	0.9331 0.9331 -	1.0717 1.0717 -	0.3653 0.1334 *p* < 0.001	2.04	0.71
BOP rate (%)	•Noodle •Processed meat •Tofu •Yogurt •Dark green vegetables •Cabbage, Napa cabbage •Carrot, Squash •Other vegetables	•Tofu •Yogurt •Cabbage, Napa cabbage •Constant term **	−0.1391 −0.1843 −0.1716 -	0.8510 0.8827 0.7850 -	1.1750 1.1329 1.2739 -	0.3209 0.1030 *p* < 0.01	2.15	9.27
PISA (mm^2^)	•Noodle •Processed meat •Tofu •Yogurt •Cabbage, Napa cabbage •Carrot, Squash	•Yogurt •Cabbage, Napa cabbage * •Constant term **	−0.1561 −0.2172 -	0.8879 0.8879 -	1.1263 1.1263 -	0.2727 0.0744 *p* < 0.05	2.29	156.9
PESA (mm^2^)	•Mushrooms	•Mushrooms * •Constant term **	0.2236 -	1.0000 -	1.0000 -	0.1992 0.0397 *p* < 0.05	2.28	258.5
PISA /PESA	•Noodle •Processed meat •Tofu •Yogurt •Dark green vegetables •Cabbage, Napa cabbage •Carrot, Squash •Other vegetables	•Yogurt •Cabbage, Napa cabbage •Constant term **	−0.1897 −0.2019 -	0.8879 0.8879 -	1.1263 1.1263 -	0.2876 0.0827 *p* < 0.01	2.14	0.101

Multiple regression analysis was calculated to predict object variables (periodontal clinical parameters) based on explanation variables (nutritional factors). Statistically significant differences (* *p* < 0.05, ** *p* < 0.01). Abbreviations: PD, probing depth; CAL, clinical attachment level; BOP, bleeding on probing; PISA, periodontal inflamed surface area; PESA, periodontal epithelial surface area; R, multiple correlation coefficient; R^2^, coefficient of determination; VIF, variance inflation factor; DW, Durbin-Watson ratio; MAE, mean absolute error.

**Table 4:** Multiple regression analysis.

	Environmental Factors							
Analysis Clinical Parameters	Correlation Coefficient |*r_s_*| > 0.15	Multiple Regression Analysis						
		*: *p* < 0.05 **: *p* < 0.01	Standardized Partial Regression Coefficient	Collinearity Statistics		R R^2^ Regression Variation	DW	MAE
				Tolerance	VIF			
Stage	N/A	N/A	-	-	-	-	-	-
Grade	•Smoking •Br Index	•Br Index * •constant term**	0.2269 -	1.0000 -	1.0000 -	0.2030 0.0412 *p* < 0.05	1.68	0.53
Number of Teeth	•Age	•Age ** •constant term**	−0.2794 -	1.0000	1.0000	0.2608 0.0680 *p* < 0.01	2.00	4.9
PD (mm)	N/A	N/A	-	-	-	-	-	-
Rate of PD 4–5 mm (%)	•Smoking	•Smoking ** •constant term **	0.3092 -	1.0000 -	1.0000 -	0.2929 0.0858 *p* < 0.01	2.18	7.1
Rate of PD ≥6 mm (%)	N/A	N/A	-	-	-	-	-	-
CAL (mm)	•Age	•Age •constant term *	0.1913 -	1.0000 -	1.0000 -	0.1616 0.0261 *p* < 0.05	2.10	0.77
BOP rate (%)	•Age •Smoking	•Age •Smoking * •constant term **	−0.1637 0.2616 -	0.9911 0.9911 -	1.0090 1.0090 -	0.2891 0.0836 *p* < 0.01	2.12	9.1
PISA (mm^2^)	•Age •Smoking	•Age •Smoking * •constant term **	−0.2656 0.2657 -	0.9911 0.9911 -	1.0090 1.0090 -	0.3687 0.1359 *p* < 0.001	2.25	150.7
PESA (mm^2^)	•Age •Smoking	•Age ** •Smoking •constant term **	−0.3591 0.1831 -	0.9911 0.9911 -	1.0090 1.0090 -	0.3959 0.1568 *p* < 0.001	2.27	247.4
PISA/PESA	•Age •Smoking	•Age •Smoking * •constant term **	−0.1654 0.2486 -	0.9911 0.9911 -	1.0090 1.0090 -	0.2776 0.0771 *p* < 0.01	2.09	0.099

Multiple regression analysis was calculated to predict object variables (periodontal clinical parameters) based on explanation variables (environmental factors). Statistically significant differences (* *p* < 0.05, ** *p* < 0.01). Abbreviations: PD, probing depth; CAL, clinical attachment level; BOP, bleeding on probing; PISA, periodontal inflamed surface area; PESA, periodontal epithelial surface area; Br Index, Brinkman index; R, multiple correlation coefficient; R^2^, coefficient of determination; VIF, variance inflation factor; DW, Durbin-Watson ratio; MAE, mean absolute error.

**Table 5:** Stratified descriptive statistics between clinical parameters and environmental factors.

	Environmental Factors							
Analysis Clinical Parameters	Multiple Regression Analysis Explanatory Variable *: *p* < 0.05 **: *p* < 0.01	Stratified Descriptive Statistics				Testing of Differences of Population Mean		
		Stratified Standard of Explanatory Variable	Numbers	Mean ± SD of Explanatory Variable	Correlation Ratio (η^2^) *: *p* < 0.05 **: *p* < 0.01	Hypothesis Testing for the Homogeneity of the Variances	Methods	*p* Value Statistical Power
Number of teeth	•Age *	<20 20≤	22 72	73.0 ± 6.07 67.8 ± 9.19	0.0632 *	*p* < 0.05	*t*-test	*p* < 0.05 * 0.6933
Rate of PD 4–5 mm (%)	•Smoking *	<2.0 2.0≤	31 63	0.48 ± 0.63 0.64 ± 0.70	0.0111	*p* = 0.49	Welch *t*-test	0.29 0.1803
BOP rate (%)	•Smoking *	<10 10≤	33 61	0.39 ± 0.50 0.69 ± 0.74	0.0434 *	*p* < 0.05	*t*-test	*p* < 0.05 * 0.5246
PISA (mm^2^)	•Smoking *	< 232 232 ≤	63 31	0.46 ± 0.59 0.84 ± 0.78	0.0695 *	*p* = 0.07	Welch *t*-test	*p* < 0.05 * 0.6479
PESA (mm^2^)	•Age *	< 1026 1026 ≤	29 65	72.8 ± 5.85 67.3 ± 9.48	0.0814 **	*p* < 0.01	*t*-test	*p* < 0.01 ** 0.8068
PISA /PESA	•Smoking *	< 0.22 0.22 ≤	63 31	0.43 ± 0.56 0.90 ± 0.79	0.1093 **	*p* < 0.05	*t*-test	*p* < 0.01 ** 0.9139

Based on the results of multiple regression analysis, environmental factors were selected and each periodontal clinical parameter was stratified by clinical standard. Correlation Ratio (η^2^) and testing of differences of population mean were calculated, respectively. Statistically significant differences (* *p* < 0.05, ** *p* < 0.01). Abbreviations: PD, probing depth; BOP, bleeding on probing; PISA, periodontal inflamed surface area; PESA, periodontal epithelial surface area.

**Table 6:** Stratified descriptive statistics between clinical parameters and nutritional factors.

	Nutritional Factors							
Analysis Clinical Parameters	Multiple Regression Analysis Explanatory Variable *: *p* < 0.05 **: *p* < 0.01	Stratified Descriptive Statistics				Testing of Differences of Population Mean		
		Stratified Standard of Explanatory Variable	Numbers	Mean ± SD of Explanatory Variable	Correlation Ratio (η^2^) *: *p* < 0.05 **: *p* < 0.01	Hypothesis Testing for the Homogeneity of the Variances	Methods	*p* Value Statistical Power
Number of teeth	•Mushrooms	<20 20≤	22 72	2.50 ± 1.19 3.81 ± 1.56	0.1241 **	*p* = 0.16	Welch *t*-test	*p* < 0.001 *** 0.9833
PD (mm)	•Yogurt *	<3.0 3.0≤	73 21	4.26 ± 2.00 2.57 ± 2.04	0.1226 **	*p* = 0.87	Welch *t*-test	*p* < 0.01 ** 0.9306
	•Cabbage, Napa cabbage *			4.75 ± 1.10 3.76 ± 1.73	0.0981 **	*p* < 0.01	*t*-test	*p* < 0.01 ** 0.8791
Rate of PD 4–5 mm (%)	•Cabbage, napa cabbage *	<2.0 2.0≤	31 63	4.68 ± 1.08 4.46 ± 1.44	0.0060	*p* = 0.09	Welch *t*-test	*p* = 0.41 0.1279
Rate of PD ≥6 mm (%)	•Cabbage, napa cabbage *	<1.0 1.0≤	61 33	4.57 ± 1.06 4.46 ± 1.68	0.0019	*p* < 0.01	*t*-test	*p* = 0.68 0.0695
CAL (mm)	•Pork, Beef, Mutton *	<4.0 4.0≤	63 31	4.29 ± 1.25 3.68 ± 0.79	0.0623 *	*p* <0.01	*t*-test	*p* < 0.05 * 0.6870
	•Dark green vegetables **			4.49 ± 1.73 3.32 ± 1.85	0.0897 **	*p* = 0.64	Welch *t*-test	*p* < 0.01 ** 0.8241
PISA (mm^2^)	•Cabbage, napa cabbage *	<232 232≤	63 31	4.67 ± 1.19 4.26 ± 1.55	0.0212	*p* = 0.08	Welch *t*-test	*p* = 0.20 0.2448
PESA (mm^2^)	•Mushrooms *	<1026 1026≤	29 65	72.8 ± 5.85 67.3 ± 9.48	0.0285	*p* < 0.01	Welch *t*-test	*p* = 0.10 0.3745

Nutritional factors were selected based on the results of multiple regression analysis and each periodontal clinical parameter was stratified by clinical standard. Correlation Ratio (η^2^) and testing of differences of population mean were calculated, respectively. Statistically significant differences (* *p* < 0.05, ** *p* < 0.01, *** *p* < 0.001). Abbreviations: PD, probing depth; CAL, clinical attachment level; PISA, periodontal inflamed surface area; PESA, periodontal epithelial surface area.

## Data Availability

The data cannot be made available because of privacy restrictions.

## Supplementary Materials

The following supporting information can be downloaded at: https://www.mdpi.com/article/10.3390/nu15020365/s1, Table S1: Questionnaire of lifestyle habits; Table S2: Results of questionnaire of lifestyle habits; Table S3: Frequency of lifestyle habits questionnaire; Table S4: Standards of diseases by blood biochemical examination items and correlation coefficients between blood examination items and nutritional factors; Table S5: Differences of clinical parameters, blood biochemical examinations, and nutritional factors between visit dentistry < 6 months and ≥ 6 months; Table S6: Correlation coefficient between IL1β in saliva and clinical parameters; Figure S1: Frequency table of habits questionnaire of nutritional factors; Figure S2: Residual plot by Breusch-Pagan & White heteroskedasticity test; Figure S3: Residual plot by Breusch−Pagan & White heteroskedasticity test.
