# Supplementary material for: Identification of Nutritional Factors to Evaluate Periodontal Clinical Parameters in Patients with Systemic Diseases
Source: Nutrients. 2023 Jan 11;15(2):365. doi: 10.3390/nu15020365 (PMC9866370; doi:10.3390/nu15020365)
Supplement: Supplementary file 1 [file nutrients-15-00365-s001.zip › nutrients-2142714-supplementary.pdf]

Table S1. Questionnaire of lifestyle habits.

Questionnaire of lifestyle habits
We ask your dietary habit in latest approximately one month.
There are a lot of items, please answer the questions with first impression.
How often did have the foods the following a) ~ w) in latest one month?
Please choose suitable one number in each item based on the frequencies and put ○, respectively.

	**Twice ≤ a day**	**Once a day**	**4~6 times a week**	**2~3 times a week**	**Once a week**	**< Once a week**	**No**
a) Rice	1	2	3	4	5	6	7
b) Bread	1	2	3	4	5	6	7
c) Noodle	1	2	3	4	5	6	7
d) Chicken (include minced meat)	1	2	3	4	5	6	7
e) Pork, Beef, Mutton (include minced meat)	1	2	3	4	5	6	7
f) Processed meat	1	2	3	4	5	6	7
g) Liver	1	2	3	4	5	6	7
h) Squid, Octopus, Shrimp, Shellfish	1	2	3	4	5	6	7
i) Fatty fish (Sardine, Mackerel, Pacific saury, Yellowtail, Herring, Eel, tuna, Fatty tuna etc.)	1	2	3	4	5	6	7
j) Nonfatty fish (Salmon, Trout, White-flesh fish, freshwater fish, skipjack tuna etc.)	1	2	3	4	5	6	7
k) Egg	1	2	3	4	5	6	7
l) Soy	1	2	3	4	5	6	7
m) Tofu, Fried tofu, Soy milk	1	2	3	4	5	6	7
n) Natto	1	2	3	4	5	6	7
o) Milk	1	2	3	4	5	6	7
p) Yogurt	1	2	3	4	5	6	7
q) Dark green vegetables (include Broccoli, bitter melon)	1	2	3	4	5	6	7

Questionnaire of lifestyle habits

We ask your dietary habit in latest approximately one month.

There are a lot of items, please answer the questions with first impression.

How often did have the foods the following a) ~ w) in latest one month?

Please choose suitable one number in each item based on the frequencies and put ○, respectively.

	**Twice ≤ a day**	**Once a day**	**4~6 times a week**	**2~3 times a week**	**Once a week**	**< Once a week**	**No**
r) Cabbage, Napa cabbage	1	2	3	4	5	6	7
s) Carrot, Squash	1	2	3	4	5	6	7
t) Radish, Turnip	1	2	3	4	5	6	7
u) Other vegetables (Onion, Burdock, lotus root etc.)	1	2	3	4	5	6	7
v) Seaweed（all kinds) (exclude soup stock)	1	2	3	4	5	6	7
w) Mushrooms (All kinds)	1	2	3	4	5	6	7

Table S2. Results of questionnaire of lifestyle habits.

	Median	Minimum	Maximum
Environmental factor			
Age	70	49	85
Smoking	0	0	2
Br index	4.5	0	1506
BMI（kg/m^2)	23.7	18.4	31.9
Nutritional factors			
Rice	6	1	7
Bread	6	1	7
Noodle	4	1	6
Chicken	4	1	7
Pork, Beef, Mutton	4	1	7
Processed meat	3	1	7
Liver	1	1	5
Squid, Octopus, Shrimp, Shellfish	2	1	7
Fatty fish	3	1	7
Nonfatty fish	4	1	7
Egg	4	1	7
Soy	4	1	7
Tofu	4	1	7
Natto	4	1	7
Milk	5	1	7
Yogurt	5	1	7
Dark green vegetables	4	1	7
Cabbage, Napa cabbage	4	1	7
Carrot, Squash	4	1	7
Radish, Turnip	3	1	7
Other vegetables	5	1	7
Seaweed	4	1	7
Mushrooms	4	1	7

The questionnaire included environmental and nutritional factors. Abbreviations: Br index, Brinkman index; BMI, body mass index.

Table S3. Frequency of lifestyle habits questionnaire.

Environmental questionnaire	**Frequency**	2		1		0	
Smoking	**Explanation**	Current smoker		Former smoker		Non smoker	
Br index	Number of cigarettes (/day) × year						
Nutritional questionnaire	**Frequency**	7	6	5	4	3	2	1
Each item	**Explanation**	Not less than twice a day	Once a day	4–6 times a week	2 or 3 times a week	Once a week	Less than once a week	No

Abbreviations: Br index, Brinkman index.

Table S4. Standards of diseases by blood biochemical examination items and correlation. Coefficients between blood examination items and nutritional factors.

**Diseases**	**Blood examination items**			**Spearman's Rank-Correlation Coefficient (r_s), 0.20 ≤ |r_s|)**	
				**Nutritional Factors**	
	**Items**	**Standard**	**Numbers**	**Items**	***:p < 0.05 **:p < 0.01**
Heart disease (n = 49) Non (n = 45)	AST (U/L) (n = 93)	30 < ≤ 30	n = 22 n = 71	Bread	−0.269 ** (n = 93)
	ALT (U/L) (n = 93)	42 < ≤ 42	n = 8 n = 86	Chicken Milk	0.207 * (n = 93) −0.216 * (n = 93)
	CK (U/L) (n = 66)	248 < ≤ 248	n = 4 n = 62	Pork, Beef, Mutton Other vegetables	−0.257 * (n = 66) −0.252 * (n = 66)
	LDH (U/L) (n = 87)	222 < ≤ 222	n = 7 n = 80	N/A	-
	NT-proBNP (pg/ml) (n = 80)	125 < ≤ 125	n = 33 n = 47	N/A	-
Dyslipidemia (n = 20) Non (n = 74)	T-Cho (mg/dL) (n = 88)	219 < ≤ 219	n = 9 n = 79	Pork, Beef, Mutton Cabbage, Napa cabbage Radish, Turnip	0.218 * (n = 88) 0.229 * (n = 88) 0.225 * (n = 88)
	LDL (mg/dL) (n = 89)	139 < ≤ 139	n = 5 n = 83	Rice Cabbage, Napa cabbage	−0.228 * (n = 89) 0.210 * (n = 89)
	TG (mg/dL) (n = 88)	149 < ≤ 149	n = 27 n = 61	N/A	-
Kidney disease (n = 35) Non (n = 59)	UN (mg/dL) (n = 93)	20 < ≤ 20	n = 23 n = 70	Tofu	0.229 * (n = 93)
	CRE (mg/dL) (n = 93)	1.07 < ≤ 1.07	n = 17 n = 76	N/A	-
	UA (mg/dL) (n =86)	7.0 < ≤ 7.0	n = 9 n = 77	N/A	-
Diabetes melli-tus (n = 28) Non (n = 66)	HbA1c (%) (n = 77)	6.2 < ≤ 6.2	n = 30 n = 47	Processed meat	0.244 * (n = 77)

Items, standards and objective numbers were shown. Dietary habit by diseases were confirmed by Spearman's rank-correlation coefficients (r_s) between blood items and nutritional factors that obtained from score of questionnaires. Student's t distribution was used to compare differences among correlation coefficients (* p < 0.05, ** p < 0.01). Abbreviations: AST, aspartate aminotransferase; ALT, alanine transaminase; CK, creatine kinase; LDH, lactate dehydrogenase; NT-proBNP, N-terminal pro-brain natriuretic peptide; T-cho, total cholesterol; LDL, low density lipoprotein; TG, triacylglycerol; UN, urea nitrogen; CRE, creatinine; UA, uric acid; HbA1c, Hemoglobin A1.

Table S5. Differences of clinical parameters, blood biochemical examinations, and nutritional factors between visit dentistry < 6 months and ≥ 6 months.

Clinical Parameters				Blood Biochemical Examinations				Nutritional Factors			
Group (n = 94)	Visit Dentistry < 6 Months	Visit Dentistry ≥ 6 Months	Differences Between Groups	Group (n = as below)	Visit Dentistry < 6 Months	Visit Dentistry ≥ 6 Months	Differences between Groups	Group (n = 94)	Visit Dentistry < 6 Months	Visit Dentistry ≥ 6 Months	Differences between Groups
Variables	(n = 49)	(n = 45)		Variables	(n = as below)			Variables	(n = 49)	(n = 45)	
Stage	2.74 ± 0.97	2.82 ± 0.91	.-	AST (U/L) (n = 93)	25.5 ± 8.93 (n = 48)	24.2 ± 8.79 (n = 45)	$p = 0.47$ Welch's *t*-test	Noodle	3.59 ± 1.59	3.47 ± 1.39	$p = 0.69$ Welch's *t*-test
Grade	2.01 ± 0.75	2.16 ± 0.67	$p = 0.52$ Welch's *t*-test	ALT (U/L) (n = 93)	25.6 ± 16.4 (n = 48)	21.9 ± 11.8 (n = 45)	$p = 0.22$ *t*-test	Pork, Beef, Mutton	4.14 ± 1.23	4.02 ± 1.08	$p = 0.08$ Welch's *t*-test
Number of Teeth	22.69 ± 5.77	22.18 ± 7.72	$p = 0.71$ Welch's *t*-test	CK (U/L) (n = 66)	114.9 ± 54.9 (n = 32)	123.1 ± 70.7 (n = 34)	$p = 0.60$ Welch's *t*-test	Processed meat	3.74 ± 1.46	3.29 ± 1.59	$p = 0.16$ Welch's *t*-test
PD (mm)	2.67 ± 0.61	2.70 ± 0.45	$p = 0.78$ Welch's *t*-test	LDH (U/L) (n = 87)	180.8 ± 31.8 (n = 46)	176.5 ± 27.4 (n = 41)	$p = 0.50$ Welch's *t*-test	Tofu	4.06 ± 1.44	4.47 ± 1.47	$p = 0.18$ Welch's *t*-test
Rate of PD 4–5 mm (%)	7.08 ± 9.00	9.24 ± 11.7	$p = 0.31$ Welch's *t*-test	NT-proBNP (pg/ml) (n = 80)	280.5 ± 346.5 (n = 39)	256.7 ± 377.2 (n = 41)	$p = 0.77$ Welch's *t*-test	Yogurt	4.10 ± 2.07	3.80 ± 2.21	$p = 0.50$ Welch's *t*-test
Rate of PD ≥ 6 mm (%)	2.45 ± 6.51	1.94 ± 3.85	$p = 0.31$ Welch's *t*-test	T-Cho (mg/dL) (n = 88)	176.7 ± 38.7 (n = 46)	165.8 ± 30.1 (n = 42)	$p = 0.14$ Welch's *t*-test	Dark green vegetables	4.45 ± 1.77	3.73 ± 1.88	$p = 0.06$ Welch's *t*-test

CAL (mm)	3.89 ± 1.15	3.89 ± 1.20	$p = 0.81$ Welch's *t*-test	LDL (mg/dL) (n = 89)	88.6 ± 28.0 (n = 46)	84.5 ± 27.3 (n = 43)	$p = 0.49$ Welch's *t*-test	Cabbage, Napa cabbage	4.55 ± 1.21	4.51 ± 1.46	$p = 0.89$ Welch's *t*-test
BOP rate (%)	15.3 ± 13.5	18.1 ± 12.7	$p = 0.31$ Welch's *t*-test	TG (mg/dL) (n = 88)	144.4 ± 113.1 (n = 48)	149.8 ± 123.8 (n = 40)	$p = 0.83$ Welch's *t*-test	Carrot, Squash	3.78 ± 1.65	3.79 ± 1.82	$p = 1.00$ Welch's *t*-test
PISA (mm^2)	207.9 ± 215.8	252.8 ± 226.7	$p = 0.33$ Welch's *t*-test	UN (mg/dL) (n = 93)	17.1 ± 5.15 (n = 49)	17.2 ± 4.35 (n = 44)	$p = 0.92$ Welch's *t*-test	Other vegetables	4.59 ± 1.70	4.31 ± 1.74	$p = 0.44$ Welch's *t*-test
PESA (mm^2)	1126.3 ± 320.7	1106.7 ± 413.3	$p = 0.80$ Welch's *t*-test	UA (mg/dL) (n =86)	5.79 ±2.88 (n = 46)	5.56 ±1.38 (n = 40)	$p = 0.64$ *t*-test	Mushrooms	3.51 ± 1.47	3.49 ± 1.70	$p = 0.95$ Welch's *t*-test
PISA /PESA	0.173 ± 0.146	0.205 ± 0.134	$p = 0.27$ Welch's *t*-test	HbA1c (%) (n = 77)	6.29 ±0.86 (n = 39)	5.96 ±0.56 (n = 38)	$p = 0.05$ *t*-test				

The differences of each parameter were compared by t-test or Welch's *t*-test between visit of dentistry groups. Student's *t* distributions were used to compare the differences. Abbreviations: n, number; PD, probing depth; CAL, clinical attachment level; BOP, bleeding on probing; PISA, periodontal inflamed surface area; PESA, periodontal epithelial surface area; AST, aspartate aminotransferase; ALT, alanine transaminase; CK, creatine kinase; LDH, lactate dehydrogenase; NT-proBNP, N-terminal pro-brain natriuretic peptide; T-cho, total cholesterol; LDL, low density lipoprotein; TG, triacylglycerol; UN, urea nitrogen; CRE, creatinine; UA, uric acid; HbA1c, Hemoglobin A1.

Table S6. Correlation coefficient between IL1β in saliva and clinical parameters.

(n = 94)	Clinical Parameters										
Saliva Components	Stage	Grade	Number of Teeth (n)	PD (mm)	Rate of PD 4–5 mm (%)	Rate of PD ≥ 6 mm (%)	CAL (mm)	BOP Rate (%)	PISA (mm^2)	PESA (mm^2)	PISA /PESA
IL1β (ng/ml)	0.096	0.130	0.058	0.381 **	0.271 *	0.317 **	0.213	0.353 **	0.299 **	0.196	0.348 **
IL1β/BCA (ng/ml)	-0.003	0.044	0.250 *	0.212	0.112	0.105	0.099	0.194	0.258 *	0.312 **	0.205

Correlation coefficients in this panel showed Spearman's rank-correlation coefficient (r_s). Eleven periodontal clinical parameters were used for the calculation of the analysis. Boxes in the matrix were painted by color in response to correlation coefficient as shown below. Student's t distribution was used to compare differences among correlation coefficients (* $p < 0.05$, ** $p < 0.01$). Abbreviations: IL1β, interleukin 1β; BCA, bicinchoninic acid; PD, probing depth; CAL, clinical attachment level; BOP, bleeding on probing; PISA, periodontal inflamed surface area; PESA, periodontal epithelial surface area.

Figure S1. Frequency table of habits questionnaire of nutritional factors.

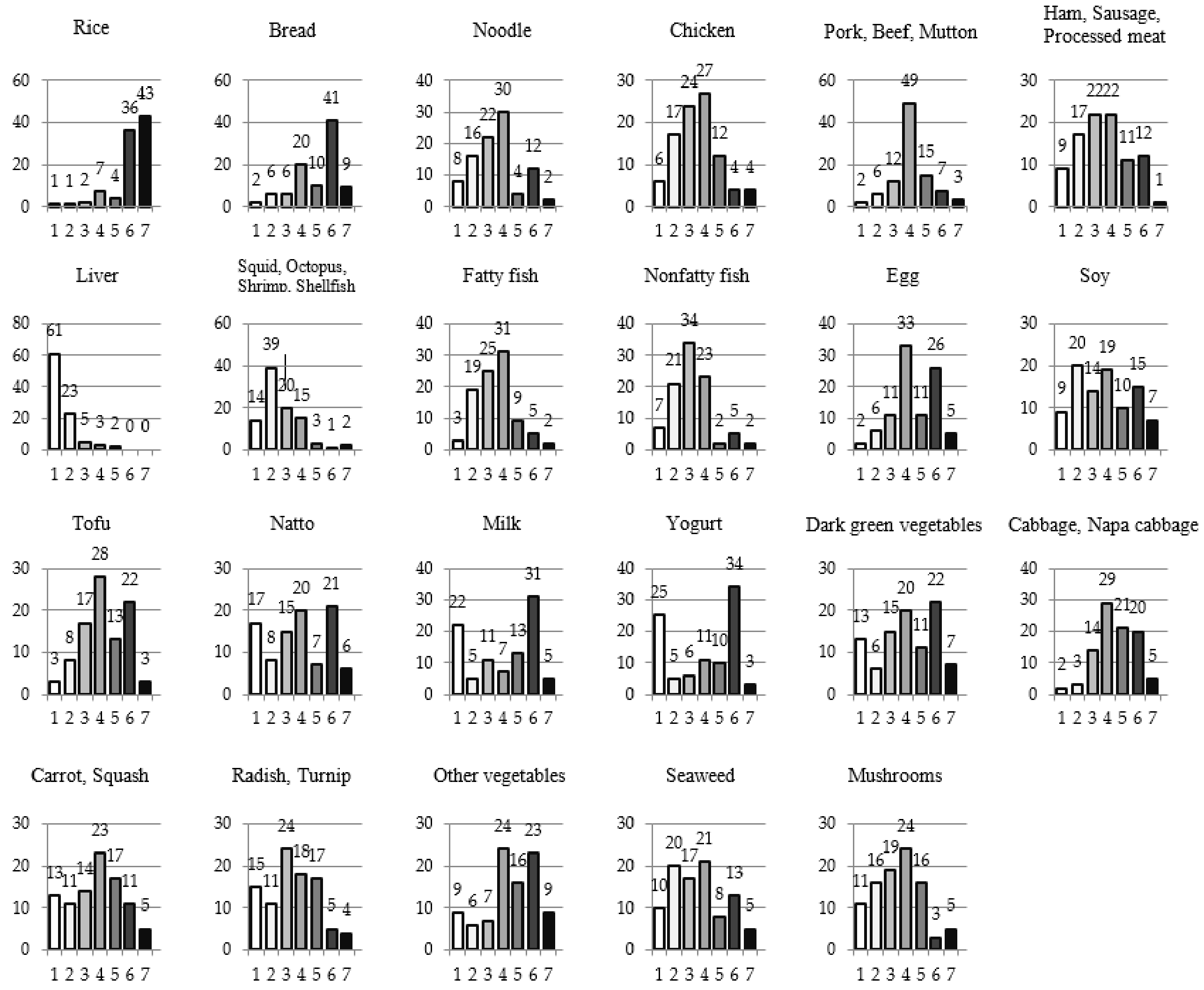

Figure S2. Residual plot by Breusch–Pagan & White heteroskedasticity test.

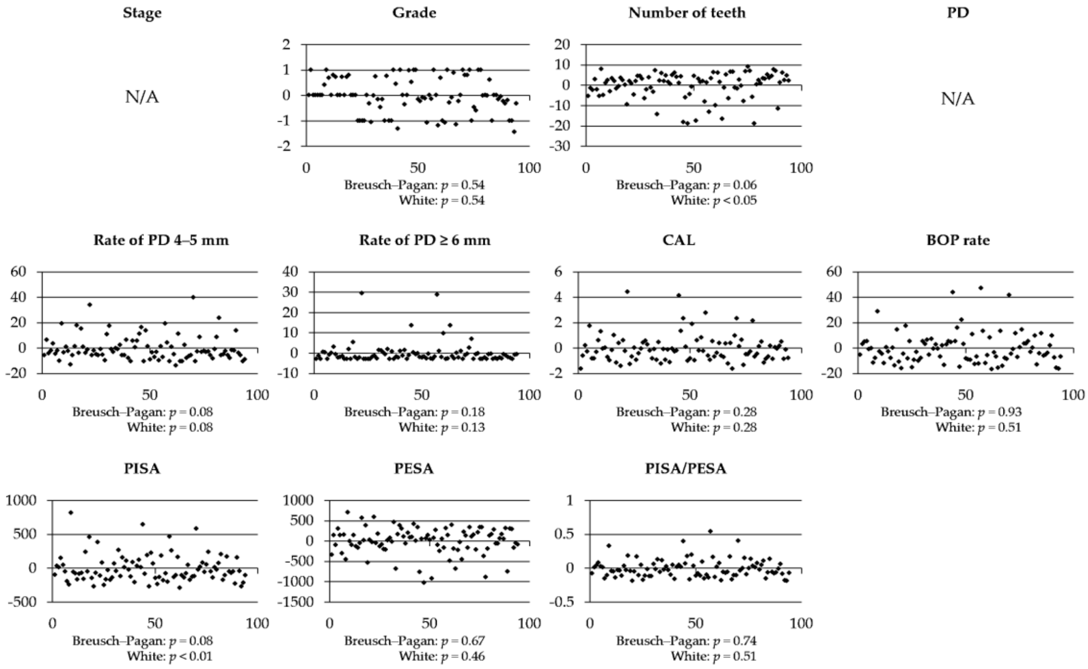

Vertical axis indicates each residual of environmental factors and horizontal axis indicates sample numbers in all graphs.

Figure S3. Residual plot by Breusch–Pagan & White heteroskedasticity test.

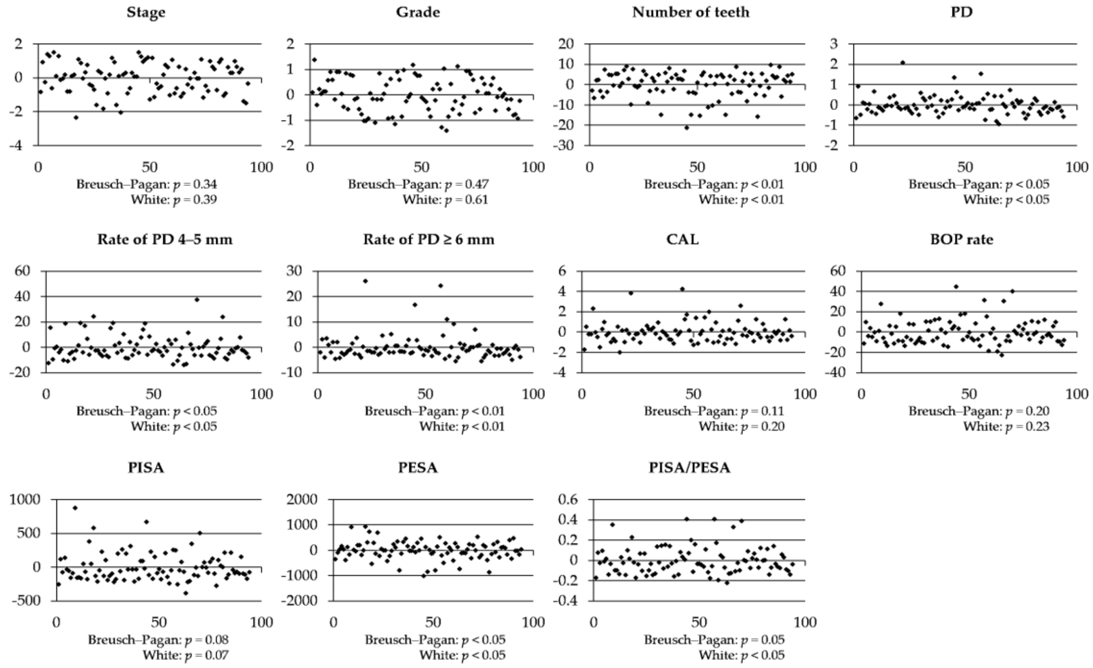

Vertical axis indicates each residual of nutritional factors and horizontal axis indicates sample numbers in all graphs.
